# A Comparison of Vitamin D Levels and Hip Fracture Severity in Elderly Patients With and Without Type 2 Diabetes Mellitus: A Retrospective Clinical Study

**DOI:** 10.7759/cureus.81574

**Published:** 2025-04-01

**Authors:** Ioannis Papaioannou, Georgia Pantazidou, Zinon Kokkalis, Neoklis Georgopoulos, Eleni Jelastopulu, Andreas G Baikousis

**Affiliations:** 1 Orthopedics and Traumatology, General Hospital of Patras, Patras, GRC; 2 Otolaryngology - Head and Neck Surgery, General Hospital of Patras, Patras, GRC; 3 Orthopedic Surgery, School of Medicine, University of Patras, Patras, GRC; 4 Endocrinology, University Hospital of Patras, Patras, GRC; 5 Public Health, University of Patras, Patras, GRC

**Keywords:** diabetes type 2, elderly patients, hip fracture severity, parathyroid level, vitamin d serum level

## Abstract

Introduction: Evidence shows that poor glycemic control and diabetes are strongly associated with poor bone quality and fragility fractures. This study was conducted to record vitamin D (VD) levels and assess hip fracture severity in elderly hip-fractured patients with and without type 2 diabetes mellitus (T2DM).

Methods: We examined 114 patients over 65 years old with low-energy hip fractures, classified as extracapsular or intracapsular. Severe fractures were defined by Garden’s classification for subcapital fractures and the AO/Orthopaedic Trauma Association classification for intertrochanteric fractures. Patients were divided into two groups: 49 with standard glycemic control (Group A) and 65 with impaired control (Group B). We measured parathyroid hormone (PTH), 25-hydroxyvitamin D, bone mineral density (BMD), hemoglobin A1c (HbA1c), serum albumin (ALB), and Homeostatic Model Assessment for Insulin Resistance (HOMA-IR).

Results: Gender and age had no significant differences. In Group A, 73.5% had osteoporosis, and 26.5% had osteopenia; in Group B, the figures were 66.2% and 33.8%, respectively (p = 0.402). VD levels were similar, with Group A averaging 10.03 ± 5.43 ng/mL and Group B 10.01 ± 5.09 ng/mL (p = 0.986). Group A's mean PTH level was 79.71 ± 57.64 ng/mL, while Group B's was 59.42 ± 45.57 ng/mL (p = 0.018). Among patients with diabetes, 60% were on oral medications, 6.2% on insulin alone, and 16.9% received a combination of treatments. Elderly diabetes patients with hip fractures, those using insulin, or those newly diagnosed with T2DM had lower VD levels. Based on regression analysis, VD is expected to decrease by 0.029 for every unit increase in PTH, concerning all the participants (95% confidence interval: 0.011-0.048). HbA1c and HOMA-IR levels showed significant differences (p < 0.001). Patients with diabetes experienced more unstable fractures (75.4% in diabetics vs. 67.3% in nondiabetics), though fracture type and severity were not statistically significant. More comminuted fractures were noted in patients on oral antidiabetic medications alongside insulin usage and those with less than five years of antidiabetic therapy. ALB levels were similar, with malnutrition prevalent in 75.5% of Group A and 75.4% of Group B patients. Patients with diabetes with malnutrition exhibited lower VD levels compared to those with normal ALB (p = 0.038). Patients with diabetes with poor glycemic control (HbA1c > 6.5%) had higher VD (10.09 ± 4.93 vs. 9.82 ± 5.57 ng/mL) and PTH (58.9 ± 49.99 vs. 50.73 ± 34.01 ng/mL) levels compared to those with adequate control (p values not significant).

Conclusions: Our study confirmed the paradox that patients with T2DM had increased BMD compared to age-adjusted nondiabetic counterparts. The difference in hip fracture severity was not statistically significant. ALB levels were similar, with malnutrition prevalent in both groups, although diabetic patients with malnutrition are correlated with lower VD levels. VD levels were similar in both groups, but PTH levels were significantly higher in nondiabetics. The relationship between T2DM and PTH remains controversial and needs further investigation. Clinicians should note that low VD levels do not accompany elevated PTH in patients with diabetes, and elevated PTH may indicate poorly controlled T2DM. VD deficiency, hypoalbuminemia, and impaired glycemic control are interconnected issues in elderly patients with low-energy hip fractures.

## Introduction

The increased life expectancy and lifestyle changes have made type 2 diabetes mellitus (T2DM) a significant public health concern globally, especially among older adults [1]. According to demographic data, the global prevalence of diabetes was estimated at 9.3% (463 million people) in 2019, and it is projected to rise to 10.2% (578 million) by 2030 and 10.9% (700 million) by 2045. Alarmingly, around 50.1% of individuals living with diabetes are unaware that they have the condition. Additionally, the global prevalence of impaired glucose tolerance was estimated at 7.5% (374 million) in 2019 and is expected to rise to 8% (454 million) by 2030 and 8.6% (548 million) by 2045 [2]. It is estimated that 20% of the elderly population worldwide suffers from T2DM, with a similar proportion remaining undiagnosed [1]. Given the notable increase in the elderly population, which accounts for 15% of the whole population (approximately 7.5 billion globally), this chronic disease's impact and its complications become apparent [1].

Poor bone mass is the most critical metabolic bone disease affecting patients with diabetes mellitus. Over the last decade, evidence has shown that individuals with T2DM are more prone to osteoporotic fractures compared to nondiabetic individuals [3]. Hip fractures pose serious consequences due to poor bone quality and osteoporosis, significantly contributing to morbidity and mortality among older patients. In the United States, more than 250,000 hip fractures occur annually in older adults, representing a serious public health issue that leads to considerable human suffering, socioeconomic burdens, and increased healthcare costs [4]. A review published in 2021 reached a consensus indicating that elderly patients with T2DM have a higher prevalence of all types of fragility fractures, especially low-energy hip fractures, compared to their nondiabetic counterparts, albeit with some debate surrounding nonvertebral fractures [5].

Current literature supports a link between vitamin D deficiency (VDD) and T2DM development. Vitamin D (VD) is thought to play a crucial role in the onset of insulin resistance and the pathogenesis of T2DM by influencing insulin sensitivity and β-cell function [6]. Our systematic review from 2021 confirmed the correlation between VDD and T2DM in elderly patients, providing strong evidence of a vicious cycle between VD deficiency and T2DM [7]. In our study, we included 34 studies, and all except four of them support the correlation of VDD and T2DM. A PubMed search with the terms "vitamin D deficiency", "diabetes mellitus type 2", and "elderly population" reveals 118 articles in the last five years. In recent years, the literature on the topic has been growing dramatically, although the results are conflicting, with the majority of the studies supporting this correlation between VDD and T2DM. The efforts are focused on establishing this correlation with particular biomolecular pathways.

This study aims to evaluate VD levels and the severity of hip fractures in elderly patients with and without T2DM. The primary objective is to determine whether glycemic control affects VD levels and the severity of hip fractures between the two groups.

## Materials and methods

This study included 114 elderly patients (over 65 years old) with and without a medical history of T2DM who sustained a low-energy hip fracture and were hospitalized between January 2021 and January 2022 in the Orthopedic Department of the General Hospital of Patras. The patient's gender and age were recorded, and age was further categorized into three groups: early elderly (65-74), middle elderly (75-84), and late elderly (85 and older).

The selection was made by simple random sampling of the eligible patients, and our study was a retrospective cohort. The inclusion criteria for the study were as follows: hip fractures resulting from low-energy trauma (excluding traffic accidents and falls from height), no history of VD supplementation, a body mass index (BMI) of less than 28, and no medical history of chronic conditions affecting bone mineral density (BMD, e.g., corticosteroid use and Paget’s disease). Additionally, patients with mild-to-severe chronic kidney failure classified as chronic kidney disease (CKD) stages 3-5 were excluded. Literature supports that VD deficiency is common in CKD stages 3-5 [8]. Patients with elevated parathyroid hormone (PTH) levels and increased serum calcium were also excluded and referred to endocrinologists for further investigation for possible primary hyperparathyroidism.

We identified two groups of patients: 49 with standard glycemic control (Group A) and 65 with impaired glycemic control (Group B), which included those with newly diagnosed prediabetes or T2DM and known long-standing T2DM. The sample of elderly patients with proximal femoral fractures, but without a history of T2DM, was initially 64 patients. However, four patients were excluded after the initial analysis of the examined parameters due to the CKD level. Notably, 11 (four with diabetes and seven with prediabetes) of the 65 patients with impaired glycemic control were diagnosed based on our findings and were subsequently referred for endocrinology consultation. On the other hand, the sample of elderly patients with proximal femoral fractures, but with a history of T2DM, was initially 56 patients. In this subgroup, two patients were excluded due to CKD level. However, in this subgroup, 11 patients were added who were found to have abnormal glycemic control. Thus, the total number of this specific group of patients was 65.

We measured the following parameters: PTH, 25-hydroxy vitamin D (25-OHVD), BMD, hemoglobin A1c (HbA1c), serum albumin (ALB) levels, and insulin resistance using the Homeostatic Model Assessment for Insulin Resistance (HOMA-IR).

The total serum concentrations of 25-OHVD and PTH were measured using radioimmunoassay, which is an in vitro assay that measures the presence of an antigen with very high sensitivity. Serum VD levels were classified according to Holick's classification [9]: severe deficiency (<10 ng/mL), mild deficiency (<20 ng/mL), inadequacy (20-29 ng/mL), and normal levels (adequacy >30 ng/mL). A PTH level of 65 pg/mL or more indicates hyperparathyroidism, especially when combined with low VD levels and typical calcium values, indicating secondary hyperparathyroidism (SHPT).

HbA1c was recorded to assess the glycemic status of each patient. For those with T2DM, adequate glycemic control was defined as an HbA1c level of less than 6.5 mg/dL, while poor control was defined as greater than 6.5 mg/dL. Among patients without a history of diabetes, glycemic status was classified as normal (HbA1c <5.7 mg/dL), prediabetes (HbA1c 5.7-6.4 mg/dL), or diabetes (HbA1c >6.5 mg/dL). For the group with T2DM, we also recorded the type of pharmaceutical therapy used and the disease duration.

The hip fractures were categorized as extracapsular (intertrochanteric) or intracapsular (subcapital). Severe subcapital fractures were defined as grades 3 or 4 according to Garden's classification [10]. In contrast, severe intertrochanteric fractures were defined as grades A2.2, A2.3, and all A3 fractures according to the AO/Orthopedic Trauma Association classification [11].

BMD was measured using dual X-ray absorptiometry (DEXA) or, in some fragile patients, with calcaneal ultrasound. BMD was classified as normal if the T-score was greater than -1.0, osteopenia if the T-score was between -1 and -2.5, and osteoporosis if the T-score was less than -2.5.

We also recorded insulin resistance using HOMA-IR levels, with normal resistance defined as HOMA-IR <1.9, moderate resistance as 1.9-2.9, and severe resistance as >2.9. For this, we recorded serum fasting insulin and glucose levels.

Finally, we measured serum ALB levels to determine whether malnutrition affected VD levels in hip-fractured patients with and without T2DM. Typical ALB values were between 3.4 and 5 g/dL, while hypoalbuminemia was defined as an ALB <3.4 g/dL.

Statistical analysis was performed using the Statistical Package for the Social Sciences version 28.0 statistical package (IBM Corp., Armonk, NY). A p value of <0.05 was considered statistically significant. Results were expressed as mean values and standard deviations (SDs) for parametric variables or as percentages for nonparametric variables. Differences between parametric variables were assessed using the unpaired t-test, while the chi-square (χ2) test was used for nonparametric variable comparisons. Relationships between different parametric variables were examined using Pearson's correlation, while nonparametric and parametric variables were compared using Spearman's correlation. Regression analysis was conducted, and normality tests were performed using the Shapiro-Wilk test and appropriate QQ plots. The Pearson correlation coefficient was used to examine the association between VD and other quantitative variables. In addition, a χ² test was performed to test for a statistically significant relationship between two categorical variables, such as the degree of fracture comminution and the patient group. For VD, where significant differences were also found, a regression model was developed to identify independent prognostic factors.

## Results

Group A comprised 14 (28.6%) male and 35 (71.4%) female patients. Only six patients (12.2%) were between 65 and 74 years old, 14 patients (28.6%) were between 75 and 84 years old, and the majority were over 85 years old (29 patients, 59.2%). The mean age was 84.71 years, with an SD of 6.74. Group B comprised 22 (33.8%) male and 43 (66.2%) female patients. Thirteen (20%), 27 (41.5%), and 25 (38.5%) patients were aged between 65 and 74, 75 and 84, and over 85, respectively. The mean age was 81.71 years, with an SD of 7.7 (Table 1). The differences in age and gender between the two groups are not statistically significant, indicating the homogeneity of our sample.

**Table 1:** Gender and age group categorization among participants in both patient groups

Parameter		Group A, n (%)	Group B, n (%)	p value
Gender	Male	14 (28.6%)	22 (33.8%)	0.549
	Female	35 (71.4%	43 (66.2%)	
Age group	65-74	6 (12.2%)	13 (20%)	0.383
	75-84	14 (28.6%)	27 (41.5%)	
	>85	29 (59.2%)	25 (38.5%)	

BMD measurements of Group A revealed osteoporosis in 73.5% and osteopenia in 26.5% of patients, while the results of Group B were 66.2% and 33.8%, respectively (Table 2). A p value of 0.402 indicates no significant difference, although the diabetic group had better BMD values than the nondiabetic group.

**Table 2:** BMD values among participants in both patient groups BMD: bone mineral density

BMD	Group A, n (%)	Group B, n (%)	p value
Osteoporosis	36 (73.5%)	43 (66.2%)	0.402
Normal BMD	0 (0%)	0 (0%)	
Osteopenia	13 (26.5%)	22 (33.8%)	

VD values were similar in both groups (10.03 ± 5.43 ng/mL in Group A vs. 10.01 ± 5.09 ng/mL in Group B), without any statistical significance (p = 0.986, Pearson correlation) (Table 3).

**Table 3:** Statistical characteristics of VD concentration among participants of both groups VD: vitamin D; SD: standard deviation

Patient’s group	Minimum value	Maximum value	Mean ± SD	p value
Group A	3.00	24.98	10.03 ± 5.43	0.986
Group B	3.00	28.20	10.01 ± 5.09	

Severe VD deficiency was more prevalent (59.2%) in Group A patients (29 patients) than in Group B (37 patients, 56.9%). The categorization of serum VD levels according to Holick’s scale is recorded in the following table (Table 4). It is worth noting that none of the patients in both groups had normal VD levels, emphasizing the prevalence of VDD. Severe VD deficiency and deficiency constitute 93.9 % of Group A and 96.9 % of Group B patients.

**Table 4:** Categorization of serum VD levels among participants of both groups VD: vitamin D

VD levels	Group A, n (%)	Group B, n (%)	p value	
			0.662	
Severe deficiency (<10 ng/mL)	29 (59.2%)	37 (59.9%)		
Deficiency (10-20 ng/mL)	17 (34.7%)	26 (40%)		
Inadequacy (20-30 ng/mL)	3 (6.1%)	2 (3.1%)		
Normal levels (>30 ng/mL)	0 (0%)	0 (0%)		

The mean PTH value of Group A was 79.71 ± 57.64 ng/mL, while in Group B, this value was 59.42 ± 45.57 ng/mL. The difference in PTH values between the two groups is statistically significant, as the p value was 0.018 (Pearson equation) (Table 5).

**Table 5:** Statistical characteristics of PTH among participants of both groups PTH: parathyroid hormone; SD: standard deviation

Patient’s group	Minimum value	Maximum value	Mean ± SD	p value
Group A	16	230.4	79.1 ± 57.64	0.018
Group B	6.5	286.6	56.42 ± 45.57	

SHPT was defined if PTH levels were above 65 pg/mL, with low VD levels and normal serum calcium levels. Patients with increased PTH levels, calcium levels, and decreased phosphorus values were excluded from the study due to the possibility of primary hyperparathyroidism and were referred for endocrinologic consultation. In our sample, SHPT was more prevalent in nondiabetic patients (Group A) with 42.9% (21 patients) in comparison with diabetic subjects (Group B) with 24.6% (16 patients). This difference was statistically significant, as the p value was 0.039, so the prevalence of SHPT is significantly higher in Group A patients. These results are summarized in Table 6.

**Table 6:** SHPT among participants of both groups SHPT: secondary hyperparathyroidism; PTH: parathyroid hormone

SHPT	Group A, n (%)	Group B, n (%)	p value
Yes (PTH ≥65 ng/mL)	21 (42.9%)	16 (24.6%)	0.039
No (PTH <65 ng/mL)	28 (57.1%)	49 (75.4%)	

HbA1c values (mean value of Group A 5.34 ± 0.19 vs. 6.36 ± 1.33 of Group B) between the two groups differed significantly, as the p value was <0.001, as was expected. This difference in HbA1c levels between the two groups is largely due to the newly diagnosed patients with extremely high HbA1c levels (Table 7).

**Table 7:** Statistical characteristics of HbA1c among participants of both groups HbA1c: hemoglobin A1c; SD: standard deviation

Patient’s group	Minimum value	Maximum value	Mean ± SD	p value
Group A	5.0	5.6	5.34 ± 0.19	<0.001
Group B	4.2	14.2	6.36 ± 1.33	

Among participants of Group B, 45 (69.2%) were found to be inadequately controlled with HbA1c levels over 6.5%, and 20 (30.8%) were adequately controlled with HbA1c levels lower than or equal to 6.4%. These results are summarized in Table 8.

**Table 8:** Glycemic regulation of T2DM patients HbA1c: hemoglobin A1c; T2DM: type 2 diabetes mellitus

Glycemic control based on HbA1c	Group B, n (%)
Adequate control (≤6.4)	20 (30.8%)
Inadequate control (>6.5)	45 (69.2%)

We further studied this patient group to evaluate VD, PTH, and fracture severity, comparing the diabetic patients with and without adequate glycemic control. Patients with poorly controlled T2DM had higher VD levels of 10.09 ± 4.93 ng/mL, higher PTH levels of 58.9 ± 49.99 ng/mL, and more unstable hip fractures (77.7%). In comparison, diabetic patients with adequate glycemic control had lower VD levels 9.82 ± 5.57 ng/mL, lower PTH levels 50.73 ± 34.01 ng/mL, and 70% of unstable hip fractures. No statistically significant difference was observed in fracture severity, VD, and PTH between the two groups, as the p values were 0.502, 0.844, and 0.507, respectively. These results are summarized in Tables 9, 10.

**Table 9:** Statistical comparison of VD and PTH levels of diabetic patients with inadequate and adequate glycemic control HbA1c: hemoglobin A1c; VD: vitamin D; PTH: parathyroid hormone; SD: standard deviation

HbA1c		n	Mean ± SD	p value
VD	Inadequate control (>6.5)	45	10.09 ± 4.93	0.844
	Adequate control (≤6.4)	20	9.82 ± 5.57	
PTH	Inadequate control (>6.5)	45	58.95 ± 49.99	0.507
	Adequate control (≤6.4)	20	50.73 ± 34.01	

**Table 10:** Statistical comparison of hip fracture severe among diabetic patients with inadequate and adequate glycemic control HbA1c: hemoglobin A1c

Fracture severity	HbA1c		Total	Χ^2^	p value
	Inadequate control (>6.5)	Adequate control (≤6.4)			
Stable	10	6	16	0.451	0.502
Unstable	35	14	49		
Total	45	20	65		

Group A had 30 (61.2%) extracapsular fractures and 19 (38.8%) intracapsular fractures, while their counterparts had 44 (67.7%) extracapsular and 21 (32.3%) intracapsular fractures without any statistical significance (p = 0.474, χ² test). The difference in hip fracture severity between the two groups was not significant (p = 0.344), although diabetics suffered more frequently from unstable fractures (75.4% vs. 67.3%) (Table 11).

**Table 11:** Characteristics of fractures among participants of both groups

Characteristics		Group A, n (%)	Group B, n (%)	p value
Fracture type	Intracapsular fracture	30 (61.2%)	44 (67.7%)	0.474
	Extracapsular fracture	19 (38.8%)	21 (32.3%)	
Fracture severity	Stable	16 (32.7%)	16 (24.6%)	0.344
	Unstable	33 (67.3%)	49 (75.4%)	

The ALB levels of both groups were similar, but without statistical significance. ALB mean value of Group A was 2.94 ± 0.56 g/dL, while in Group B was 2.94 ± 0.53 g/dL) (Table 12).

**Table 12:** Albumin levels among participants of both groups SD: standard deviation

Patient’s group	Minimum value	Maximum value	Mean ± SD
Group A	1.50	4.40	2.94 ± 0.56
Group B	1.60	3.90	2.94 ± 0.53

It is worth noting that hypoalbuminemia and, subsequently, malnutrition were prevalent in the majority of subjects in both groups. More specifically, 75.5% of Group A and 75.4% of Group B were characterized by diminished ALB levels. The following table summarizes these results in detail (Table 13).

**Table 13:** Distribution of albumin levels among participants of both groups

Albumin	Group A, n (%)	Group B, n (%)
Normal values (3.4-5 g/dL)	12 (24.5%)	16 (24.6%)
Decreased (<3.4 g/dL)	37 (75.5%)	49 (75.4%)

Inductive statistical analysis with t-test evaluation of Group B participants revealed significantly reduced values of VD (12.3 ± 5.45 ng/mL vs. 9.26 ± 4.8 ng/mL) among patients with diminished levels of serum ALB compared with those with normal values of ALB. This difference was statistically significant, as the p value was 0.038, although this difference was not observed in nondiabetic patients (Group A) (Table 14).

**Table 14:** VD variations of diabetic patients based on serum albumin levels in Group B patients VD: vitamin D; SD: standard deviation

Albumin levels	VD		
	n	Mean ± SD	p value
Normal values (3.4-5 g/dL)	16	12.3 ± 5.45	0.038
Reduced values (<3.4 g/dL)	49	9.26 ± 4.8	

The HOMA-IR values (mean value of Group A 1.62 ± 1.06 vs. 2.63 ± 2.58 of Group B) between the two groups differed significantly, as the p value was found to be 0.011, as was also expected (Table 15). HOMA-IR is calculated based on glucose and fasting insulin levels. As expected, patients (Group A) with impaired glucose levels had significantly higher levels of HOMA-IR.

**Table 15:** Statistical characteristics of insulin resistance among participants of both groups

Patient’s group	Minimum value	Maximum value	Mean value	Standard deviation	p value
Group A	0.12	4.11	1.62	1.06	0.011
Group B	0.17	16.94	2.63	2.58	

In Table 16, we recorded the distribution of insulin resistance levels among participants of both groups based on HOMA-IR levels. Normal insulin resistance was defined as HOMA-IR less than 1.9, moderate insulin resistance as HOMA-IR between 1.9 and 2.9, and abnormal as HOMA-IR more than 2.9 (Table 16). This association has no statistical significance, indicating that all participants should be monitored for T2DM prevention.

**Table 16:** Distribution of HOMA-IR levels between both groups HOMA-IR: homeostatic model assessment-insulin resistance

HOMA-IR	Group A, n (%)	Group B, n (%)	p value
Normal HOMA-IR	27 (55.1%)	27 (41.5%)	0.207
Moderate HOMA-IR	13 (26.5%)	17 (26.2%)	
Severe HOMA-IR	9 (18.4%)	21 (32.3%)	

We performed further statistical analysis based on the Pearson equation, and we found statistical significance when we correlated PTH and VD levels among participants of Group A (p = 0.014). This correlation was not disclosed in Group B patients; the p value was 0.165. Further regression analysis revealed that this statistical significance between VD and PTH concerns all the participants and not only Group A patients. Particularly, it is expected that VD will be reduced by 0.029 for every unit of PTH increase (95% confidence interval, CI: 0.011-0.048).

Concerning the statistical characteristics of diabetic patients, the analysis disclosed the following data. Among diabetic patients, 60% (39 patients) were treated with antidiabetic pills, 6.2% (four patients) with insulin alone, 16.9% (11 patients) with a combination of pills and insulin, and 16.9% (11 patients) were newly diagnosed, and treatment was determined by endocrinologists. Forty percent (26 patients) of diabetic patients were treated with only one antidiabetic agent, 43.1% (28 patients) were treated with more than one pharmaceutical agent, and 16.9% (11 patients) were newly diagnosed (Table 17).

**Table 17:** Type of antidiabetic therapy and number of agents used in Group B patients

Parameter		n (%)
Type of antidiabetic pharmaceutical therapy	Antidiabetic pills	39 (60%)
	Insulin	4 (6.2%)
	Combination (pills and insulin)	11 (16.9%)
	New diagnosis	11 (16.9%)
Number of antidiabetic agents	One	26 (40%)
	More than one	28 (43.1%)
	New diagnosis	11 (16.9%)

Most diabetic patients suffered from T2DM for more than five years (63.07%, 41 patients), while 20% (13 patients) had T2DM for less than five years (Table 18).

**Table 18:** Display of duration of T2DM among Group B participants T2DM: type 2 diabetes mellitus

Duration of T2DM	n (%)
<5 years	13 (20%)
>5 years	41 (63.1%)
New diagnosis	11 (16.9%)

According to the statistical analysis, VD levels and hip fracture severity were not significantly different. However, lower levels of VD were noticed in newly diagnosed patients (VD 8.05 ± 4.33) and those with insulin intake as monotherapy (VD 7.6 ± 2.6). These subgroups may have the worst glycemic regulation.

Diabetic patients with a T2DM duration of less than five years suffered from more severe hip fracture patterns (84.6% unstable fractures). Finally, patients who received per os antidiabetic drugs, accompanied by insulin intake (90.9% unstable fractures), also had more comminuted fractures. It is worth noting that these findings had no statistical significance.

## Discussion

There is a consensus that VDD is closely related to T2DM [7], particularly in elderly patients with poorly controlled diabetes. VDD increases insulin resistance, decreases insulin sensitivity, and impairs beta-cell function by affecting the molecular repair mechanisms of these cells. Conversely, adequate VD levels reduce oxidative stress, minimize inflammation, and enhance insulin signal transduction [12].

Our study compared two groups and found similar diminished VD levels in both. None of the participants had normal VD levels, and deficiency was prevalent in both groups. Despite differences in glycemic status, the participants shared several characteristics that contributed to these results regarding VD levels. All patients were elderly with minimal sun exposure and age-related skin changes. Additionally, liver and kidney function, which are often impaired in elderly patients, negatively impact the synthesis of VD. Poor nutrition, characterized by low serum ALB levels and the fact that food and milk in Greece are not adequately fortified with VD, further exacerbates VDD [13].

To our knowledge, no studies exist in the literature comparing VD levels among diabetic and nondiabetic elderly patients who have suffered hip fractures. Notably, lower VD levels were observed in newly diagnosed patients with T2DM and those using insulin as monotherapy. These subgroups tend to have poorer glycemic regulation, and poorly controlled diabetes is correlated with more severe VDD [14]. Greece, a Mediterranean country with abundant sunshine throughout most of the year, often leads to an underestimation of VD deficiency. However, evidence indicates that severe VDD is prevalent among the elderly in Greece, and this deficiency is not limited to hip-fractured patients [15].

Given that VD levels were found to be similar in both groups, we expected PTH levels to be comparable as well. However, the T2DM group exhibited statistically significantly lower PTH levels. Current studies show a direct suppressive effect of high glucose concentrations on PTH secretion, while higher PTH levels within the normal range may enhance beta-cell function [16]. Human beta cells have been shown to express PTH-1 receptors, supporting the correlation between PTH levels and beta-cell function [17]. This relationship is reinforced by recent experimental studies demonstrating beta-cell regeneration following partial pancreatectomy and administration of PTH-related peptides [18].

A more straightforward explanation for the lower PTH levels in patients with T2DM could be that inflammation induced by hyperglycemia hinders PTH production [19]. Additionally, diabetes-related microangiopathy might contribute to parathyroid gland dysfunction [20]. Statistical analysis showed statistical significance when we correlated PTH and VD levels among participants of Group A (p = 0.014). This correlation was not disclosed in Group B patients. The dysregulation of PTH due to hyperglycemia could play a role in this finding and warrants further investigation. However, further regression analysis showed that VD is expected to decrease by 0.029 for every unit increase in PTH, concerning all the participants (95% CI: 0.011-0.048). Diabetic patients with poor glycemic control (HbA1c > 6.5%) had higher VD (10.09 ± 4.93 vs. 9.82 ± 5.57 ng/mL) and PTH (58.9 ± 49.99 vs. 50.73 ± 34.01 ng/mL) levels compared to those with adequate control (p values were not statistically significant). From the perspective of optimizing bone health, clinicians should note that low PTH levels do not necessarily correlate with adequate VD levels in elderly patients with T2DM, while elevated PTH may indicate poorly controlled T2DM [21].

Despite the small sample size, our study confirmed that T2DM patients are associated with higher BMD, but remain at an increased risk for fractures. This paradox, supported by existing literature [22], should be considered by physicians when developing fracture prevention strategies. Increased BMI affects BMD measurements, and individuals with higher weight, such as those with T2DM, have higher BMD compared to their age-matched counterparts. Based on this paradox, many studies conclude that BMD is not sensitive enough to assess the risk for fragility fractures in T2DM patients [22]. On the other hand, T2DM patients fractured more frequently because they cannot repair microcracks of the bone due to inadequate bone turnover [23]. The trabecular bone score (TBS) and the revised Fracture Risk Assessment Tool (FRAX) score, the FRAX plus published in 2023 [24], are crucial in achieving this goal since the classic FRAX score underestimates the fracture risk in diabetic patients. FRAX plus takes into consideration all the following features: recency of osteoporotic fracture, higher than average exposure to oral glucocorticoids, TBS, number of falls in the previous year, duration of T2DM, and concurrent information on lumbar spine BMD and hip axis length [24].

Bone fragility should be recognized as a new complication of T2DM, especially in elderly patients. These individuals are particularly vulnerable to T2DM-related bone fragility due to additional factors, such as senile osteoporosis, severe VD deficiency [7], comorbidities, insulin use, and diabetes-related complications [25], particularly diabetic neuropathy and retinopathy, which predispose them to falls [15]. Furthermore, it is known that poorly controlled diabetics often present with lower calcium ion levels and insufficient PTH secretion [25]. Unrecognized T2DM should be investigated to eliminate an additional risk factor for poor bone quality and the well-known complications associated with T2DM [6,13].

Our study found that approximately 75% of patients in both groups had hypoalbuminemia. As previously reported, all participants exhibited abnormal VD levels. We conducted a statistical analysis to examine the correlation between VD and ALB levels. In Group B, the VD levels were significantly lower among patients with reduced serum ALB than those with normal ALB levels. This correlation was statistically significant.

Current literature supports the connection between VD and ALB levels, and the underlying mechanisms are still being investigated [26]. Malnutrition is associated with low serum ALB and VD levels. One potential explanation is that about 75% of VD is bound to vitamin D-binding protein (DBP), 15% is bound to ALB, and less than 1% is free VD [27]. It is important to note that vitamin DBP is part of the ALB gene family, including human serum ALB and alpha-fetoprotein [28].

Experimental studies have demonstrated that inadequate protein and energy intake can reduce circulating DBP concentrations [29]. Additionally, in vivo studies suggest that DBP and ALB play a critical role in influencing the availability of 25-OHVD to 1-alpha-hydroxylase, the enzyme responsible for converting 25-OHVD to 1,25-dihydroxyvitamin D [30]. From a clinical perspective, low ALB levels should prompt physicians to consider the possibility of malnutrition and, consequently, lower VD levels. Nutritional status significantly impacts functional outcomes and mortality rates after hip fractures, while, in addition to serum ALB, lymphocyte count may also indicate a patient’s nutritional status [31].

The difference in hip fracture severity between the two groups was not statistically significant (p = 0.344). However, individuals with diabetes experienced unstable fractures more frequently (75.4% vs. 67.3%). Among diabetic patients, those with a relatively short duration of the disease (fewer than five years since their T2DM diagnosis) and those taking oral antidiabetic medications along with insulin were more likely to suffer unstable fractures. This suggests that these diabetic patients may be less well-controlled and could experience multiple hypoglycemic episodes, leading to falls. The severity of hip fractures is closely associated with patients’ mortality rate and functional outcome. Unstable and severe comminuted hip fractures are linked to higher mortality rates and poorer functional status [32]. Notably, the functional status before the injury is the only independent predictor of long-term functional outcomes and mortality.

Our study corroborated that diabetic patients are more prone to experience displaced hip fractures. Interestingly, while diabetic patients generally correlate with higher BMD, this observation presents a paradox that clinicians should be aware that BMD alone is not a reliable measure of bone quality in diabetic patients, necessitating additional diagnostic tools.

In contrast, our study revealed that nondiabetic patients had lower BMD values and less severe hip fractures. This finding aligns with existing literature [33]. A possible explanation is that as BMD decreases, less mechanical force is required to cause hip fractures, which may not produce significant displacement or unstable fracture patterns.

Limitations

The results of our paper should be interpreted carefully due to the existing limitations of the study. First, our study was a retrospective one, and this fact may affect the quality of the data compared to a prospective study. The second limitation is our study's relatively small sample size and the difficulty of exclusively recognizing individuals with our inclusion criteria from one trauma center. The third limitation is the fact that this study was performed in one trauma center in Greece, and ethnic or racial differences in VD levels should be considered. Therefore, the generalization of the study results should be restricted. The fourth limitation is the method of BMD measurement. In some highly fragile patients, DEXA could not be performed, and we proceeded with calcaneal ultrasound BMD measurement. Measuring BMD using the quantitative ultrasound method cannot replace DEXA; however, it is a reliable tool for measuring osteoporosis in cases where DEXA is not feasible. Fifth, we defined prediabetes and T2DM according to HbA1c. However, prediabetes and diabetes can also be diagnosed with fasting glucose and glucose tolerance tests. These different indicators might have identified somewhat different subjects for inclusion.

Strengths

Despite its many limitations, the present study has important strengths. To the author’s knowledge, this is the first retrospective study to record the VD levels among diabetic elderly patients with hip fractures. Furthermore, this paper constitutes a unique attempt to compare diabetic and nondiabetic elderly patients with hip fractures regarding VD, PTH, hip fracture severity, serum ALB, and BMD. Finally, the number of participants in our study was adequate, considering that the survey was conducted in a single trauma center and the multiple exclusion criteria. More extensive multicenter worldwide studies, in collaboration with the fragility fracture network, will provide strong evidence.

## Conclusions

Our study confirmed the paradox of increased BMD in patients with T2DM compared with their age-adjusted nondiabetic counterparts. VD levels were similar in both groups; however, PTH was statistically significantly higher in the nondiabetic group. Clinicians should be aware of the second paradox that arises from our study: low VD levels do not accompany elevated PTH in diabetic patients, and elevated PTH may indicate poorly controlled T2DM. Hip fracture severity differences between the two groups were not statistically significant, although diabetic patients experienced unstable fractures more frequently. Malnutrition was prevalent in both groups, although decreased ALB levels in diabetic patients were correlated with reduced VD levels in comparison to diabetic patients with normal ALB levels. Decreased ALB should alert physicians to possible malnutrition and subsequent lower VD levels. VD deficiency, hypoalbuminemia, and impaired glycemic status consist of elements of a circle, which requires a holistic management of an elderly patient with a low-energy hip fracture. Among diabetic elderly patients with hip fractures, those with insulin intake and newly diagnosed T2DM were correlated with lower VD levels. In contrast, more comminuted fractures were prevalent among patients with less than five years of antidiabetic therapy and those who receive per os antidiabetic drugs, accompanied by insulin intake. The third and final paradox of this study is that despite the increased BMD values of diabetic patients, these hip-fractured elderly patients are more prone to experience displaced hip fractures and suffer all the consequences. VD and protein supplementation are mandatory in both groups, while antiosteoporotic treatment is of utmost importance to reduce fragility fractures. Physicians should be aware of all the specific methods related to diabetic patients to eliminate misdiagnosis. Malnutrition has a very high prevalence among the elderly, while more studies are needed to confirm if VD supplementation improves the glycemic profile of elderly diabetic and nondiabetic patients. 
